# Measuring Sedimentation Profiles for Nanoparticle Characterization through a Square Spiral Resonator Sensor

**DOI:** 10.3390/s24092735

**Published:** 2024-04-25

**Authors:** Miguel Monteagudo Honrubia, Gianluca Caposciutti, Francisco Javier Herraiz-Martínez, Javier Matanza Domingo, Bernardo Tellini, Romano Giannetti

**Affiliations:** 1 Institute for Research in Technology, ICAI School of Engineering, Comillas Pontifical University, 28015 Madrid, Spain; mmonteagudo@comillas.edu (M.M.H.); fjherraiz@icai.comillas.edu (F.J.H.-M.); jmatanza@comillas.edu (J.M.D.); 2 Department of Energy, Systems, Territory and Construction Engineering, University of Pisa, 56122 Pisa, Italy; gianluca.caposciutti@unipi.it (G.C.); bernardo.tellini@unipi.it (B.T.)

**Keywords:** microwave sensors, permittivity, metallic nanoparticles, sedimentation

## Abstract

Metallic nanoscale particles attract a growing interest in several fields, thanks to their unique bonding characteristics; applications are appearing in the literature in the fields of, for example, sensor coatings and biochemical compound detection. However, the controlled fabrication of such nanopowders is often cumbersome, especially because their characterization is normally slow, involving procedures such as electron microscopy. On the other hand, microwave sensors based on near-field effects on materials are being developed with high sensitivity and show promising characteristics. In this paper, the authors show how a microwave sensor based on a Square Spiral Resonator can be used to characterize paraffin dispersions of nanoparticles conveniently and cost-effectively.

## 1. Introduction

### 1.1. The Challenge of Nanoparticle Characterization

Nanoparticles (NPs) are ultrafine particles with an equivalent size in the order of $10^{-9}$ m. More concretely, particles ranging from 1 to 100 nm are usually referred to as nanoparticles. Powders of such dimensions show easy bonding with the contacting materials, large surface area, low melting point, and peculiar electromagnetic and optical properties. For these reasons, NPs are appealing for use as a sensing element and for controlling the characteristics of the materials once deposited or dispersed [1].

For example, metal nanopowders find significant utility as sensing elements in biomedical sensors, leveraging phenomena such as surface plasmon resonance [2,3]. In particular, metal NPs can selectively form bonds with enzymes, antibodies, or proteins of different structures. These bonds alter the surface light-absorption properties, enabling the precise measurement of the characteristics of the bound material with heightened sensitivity and specificity [4,5]. Metal NPs are also utilized in the thermal and photo-thermal treatment of cells [3]. For instance, the deposition of stable NPs over a specific target improves the local irradiation capability and allows for better control over laser treatments [6]. Furthermore, these particles can be exploited as coating agents for their antimicrobial properties [7] and, more broadly, enhance the mechanical characteristics and chemical resistance of surfaces [8,9]. More recently, nano-sized ${\mathrm{LiMn}}_{2}$$O_{4}$ was utilized for the realization of the battery’s cathodes [10]. Specifically, using such nanoparticles increases the mechanical flexibility of the system and its electrical capacity due to the extensive surface area provided by the NPs.

The characteristics of the particles are mainly determined during their fabrication. In general, these processes can be divided into bottom-up and top-down ones. In the former, single or small clusters of molecules are utilized as the building block for producing NPs with controlled shape and size. For example, the chemical vapor deposition method is widely used for this purpose [11]. Generally, a gas reactant passes through a substrate where the nanoparticles layers are formed due to heterogenous reactions [12]. Although the bottom-up methods can provide fine control of the process outcome, these are generally energy- and cost-demanding, producing environmentally harmful by-products. Other techniques can produce small particles from a bulk material (i.e., top-down approaches). For example, laser ablation allows for the production of metal NPs with sizes in the order of tens of nanometers, employing high-power laser pulses with nanosecond duration [13]. Similar approaches, such as mechanical milling, sputtering, or thermal evaporation [14], take advantage of the lower cost and hardware complexity but are ineffective for the precise control of particle shapes and dimensions or for the manufacture of extremely small elements. Other methods, such as exploding wire [15], enable a reduction in thermal losses through localized power dissipation, possibly enhancing productivity and energy efficiency, and the ability to produce multicomponent nanostructures, even when immiscible with standard techniques [16,17].

In this framework, nanoparticle characterization is of paramount importance in evaluating the outcome of production processes in a timely and convenient way, adjusting the fabrication parameters in a tight loop to obtain the desired characteristics. However, exploring the nanoscale to determine properties such as particle size, size distribution, surface charge, or shape is not a trivial task [18]. In an extensive review, Mourdikoudis et al. summarized the whole toolbox of techniques to characterize nanoparticles [19]. For example, the determination of key size parameters is commonly achieved through microscopy techniques, such as scanning electron microscopy (SEM) or transmission electron microscopy (TEM). These methods reconstruct images of the particles, enabling the assessment of dimensional distributions, including circularity and equivalent diameter via image analysis methods. In contrast, spectroscopy techniques, such as dynamic laser scattering, rely on the interaction between a laser beam and the Brownian motion of the particles to obtain information on the equivalent diameter of particles [20]. In addition, other techniques, such as energy-dispersive X-ray spectroscopy (EDS), can be employed to identify the chemical elements present in products. An alternative approach involves the analysis of NP sedimentation in liquid dispersions, where the interplay of diffusion and gravity forces determines the behavior and characteristics of NPs. Thus, by studying the dynamic of the dispersed NP, their size and density can be estimated, e.g., in combination with digital imagery techniques [21] and proper modeling approaches.

Unfortunately, these characterization methods require either time-consuming protocols or complex instrumentation, with a significant share of manual interventions, causing long delays among runs of the fabrication process. Therefore, new analysis procedures are required to assess the NPs’ features with lower costs. This article proposes a Microwave (MW) sensor based on Spiral Square Resonators (SSR) to measure the dynamic changes in the dielectric properties of NP dispersions due to sedimentation. This novel approach enables a fast and straightforward measuring protocol that requires low volumes, simplifying the characterization of NPs with minimal waste of fabricated samples. This research shows the potential of low-cost SSR sensors to monitor the sedimentation process of NPs with diverse chemical compositions and initial solution concentrations.

### 1.2. Microwave Sensor

MW sensors, particularly Split-Ring Resonator (SRR) sensors, provide a flexible and cost-effective method for characterizing materials. Regardless of the design, the sensor operates as a notch filter represented by a high-frequency RLC resonant circuit with a high-quality factor. Since the resonator capacitance is related to the medium permittivity, any medium change, such as the presence of the target material, would lead to a shift in the resonance frequency [22]. The variation in the resonance frequency depends on the dielectric characteristic of the target material; larger permittivity would lead to larger frequency shifts. On the other hand, the topology and dimensions of the SRR define the resonance frequency; therefore, the sensor design can be tuned to work for different applications in a specific range of frequencies [23]. Spiral resonator design allows smaller electrical dimensions, enhancing the quality factor and the sensitivity [24].

**Figure 1:**
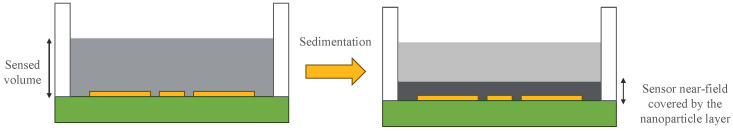
Sedimentation process in the sensor.

Moreover, the number of resonators is not limited to one, and several resonators in a symmetric configuration can improve the resonance. These sensors have proven potential for numerous applications, including the dielectric characterization of thin layers since the miniaturizing resonators will confine the electric field in the near space surrounding [25]. This paper proposes a Square Spiral Resonator sensor in the microwave regime to measure nanoparticle sedimentation. The deposition of nanoparticles will create a uniform layer in the sensor surface, modifying the capacitance of the equivalent resonant circuit, thereby changing the resonance frequency along the sedimentation time (Figure 1). The maximum frequency shift and the convergence time to the “layer” resonance frequency are related to the dispersion concentration and the sedimentation dynamics of different nanoparticles.

## 2. Methods

### 2.1. Sedimentation of Nanoparticles

The sedimentation of solid particles in a liquid medium mainly depends on the density and shape of the solid elements and on the viscosity, density, and temperature of the liquid. With the purpose of providing a simple but reliable description of the sedimentation and of introducing the method exploited for NPs study, we consider the contribution of the Brownian and the gravitational forces in a mono-dimensional system (i.e., along the *x*-axis) over time *t*. Therefore, the concentration *c* of the disperse phase of solid particles behaves according to the Mason–Weaver Equation (1) [21,26].

(1) $$\frac{\partial c}{\partial t}=D\frac{\partial^{2}c}{\partial x^{2}}+v\frac{\partial c}{\partial x}$$

where *D* and *v* are experimental coefficients depending on the particle size, density, and liquid physical characteristics. In particular, $D=k_{b}T/F$ is the diffusion coefficient depending on the fluid temperature *T*, the Boltzman constant $k_{b}$, and the frictional coefficient *F*. Moreover, $v=m_{b}g/F$ is the sedimentation velocity that depends on the buoyancy mass, $m_{b}$; on the gravitational acceleration *g*; and on *F*. For spherical particles with radius *r* and density ρ, and a liquid with viscosity η and density $\rho_{L}$, F=6πηr and $m_{b}=\frac{4}{3}\pi r^{3}\left(\rho -\rho_{L}\right)$ [21]. We further assume that $\frac{\partial c}{\partial x}+\frac{v}{D}c=0$ at x=0 and $x=x_{max}$, and $c\left(t=0,x\right)=c_{0}$, where $x_{max}$ is the maximum system length and $c_{0}$ is the initial concentration). The solution of Equation (1) shows that *c* changes with *t*, finally achieving an equilibrium distribution for t→∞ [21]. This result can be interpreted as the growth of a particles layer on the sensor surface, thus increasing the average permittivity in the sensitive volume of the sensor and hence decreasing the value of the measured resonance frequency *f*. The evolution of *f* vs. *t* depends on the sedimentation dynamic, and it is peculiar to the particle’s material, dimension, and initial concentration. Therefore, by monitoring such behavior we can carry out information on the properties of the solid dispersed phase. Although the model in Equation (1) does not consider particle clustering processes, which could influence the sedimentation dynamic and hence the attended readout [27], it offers a proof-of-concept of the operating principle of the sensor and can be used as a basis for a discussion about the obtained results.

### 2.2. Nanoparticle Production

**Figure 2:**
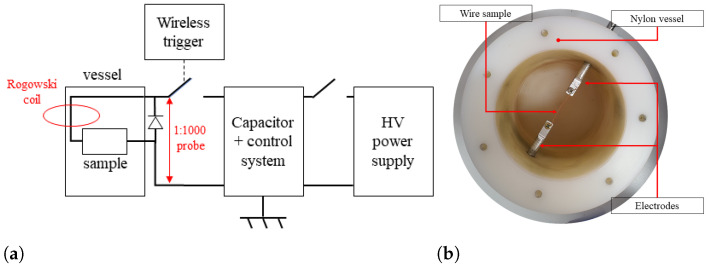
The scheme of the exploding wire setup (**a**) and a picture of the vessel (**b**) utilized for NPs fabrication.

Copper- and iron-based nanoparticles were produced at the facility of the University of Pisa. We adopted a wire explosion method for NPs fabrication, and we used a microscopy-based technique to assess the main size characteristics of the products. The NPs are produced by flowing a current in the order of 1 × 10^7^ A/$m^{2}$ to 1 × 10^9^ A/$m^{2}$ through a cylindrical copper or iron wire with a diameter of 1 mm and 30 mm length. The current is provided by a 765 μF capacitor, with a voltage limit of 10 kV, loaded through a High Voltage (HV) power supply. Voltage and current are monitored during the process by utilizing a 1:1000 dedicated voltage probe, a calibrated Rogowski coil, and an oscilloscope (Lecroy W wave Pro 725Zi, Teledyne LeCroy, Italy) with four 8-bit boards and 2.5 GHz bandwidth. Due to the high current density, the process enables adiabatic heating of the sample, which rapidly vaporizes to form small clusters of molecules, further condensing in contact with the surrounding medium, thus producing the NPs. To collect the product and control the explosion environment, we utilized distilled water as a medium surrounding the exploding wire. A picture and a scheme of the utilized setup are shown in Figure 2.

**Figure 3:**
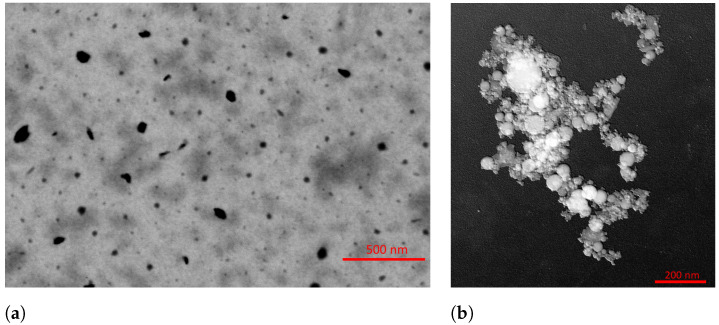
Sample images of Cu-NPs at 160,000×magnification obtained with a bright field STEM detector (**a**) and of Fe-NPs at 200,000× magnification obtained with a STEM detector (**b**).

We produced the copper-based NPs (Cu-NPs) and iron-based NPs (Fe-NPs) separately. A single sample was subjected to explosion, and the resulting water containing the nanoparticles was collected in a larger container. Subsequently, the explosion vessel and electrodes were accurately cleaned with deionized water to minimize any contamination between the test runs. This procedure was iterated ten times to ensure the fabrication of an average representative sample. The nanoparticles were ultimately extracted from the liquid via a low-temperature evaporation process. Therefore, they were diluted in deionized water, deposited on dedicated samples, and prepared for microscopy analyses (Figure 3).

**Figure 4:**
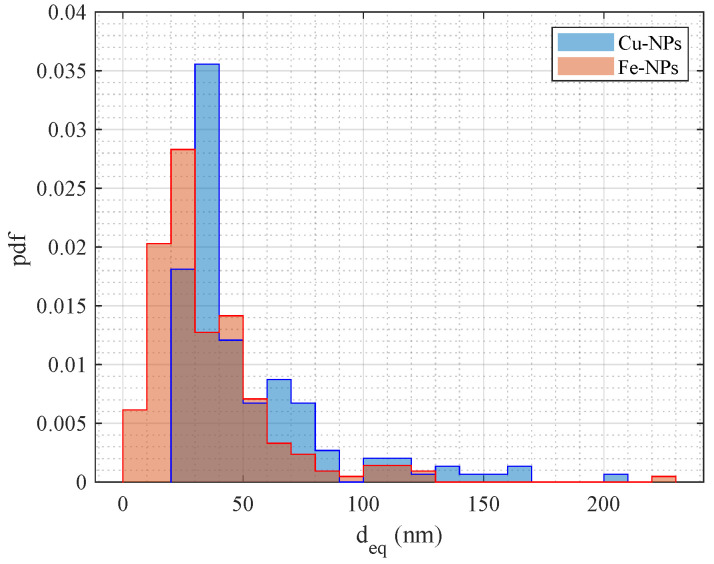
Dimensional distribution of the equivalent diameter, $d_{eq}$, of the NPs analyzed samples.

To provide an indication regarding the dimensions of the smaller synthesized elements, we examined the images at a magnification of 160,000× and 200,000× via a particle counting. The copper-based NPs were analyzed using the FEG-SEM (FEI QUANTA 450 ESEM-FEG, Thermo Fisher Scientific, Hillsboro, OR, USA), and the HR FEG-TEM (JEOL JEM-F200, Jeol Ltd., Tokyo, Japan) was used for the iron-based NPs. These instruments are available at the Center for Instrument Sharing of the University of Pisa (CISUP). The results regarding the particle size distribution (evaluated as probability density functions, pdf, of $d_{eq}$) are shown in Figure 4. Finally, the minimum, average, median, and maximum equivalent diameter of the sampled NPs, analyzed at a magnification within 160,000× and 200,000×, are reported in Table 1.

The EDS analysis was also performed, confirming the production of Cu-based and Fe-based nanoparticles. However, the presence of oxygen, along with copper and iron, respectively, possibly indicates an oxidized state of the products. Metallic impurities, mainly aluminum, are present in minor proportion due to the electrodes erosion.

### 2.3. Sensor Structure

**Figure 5:**
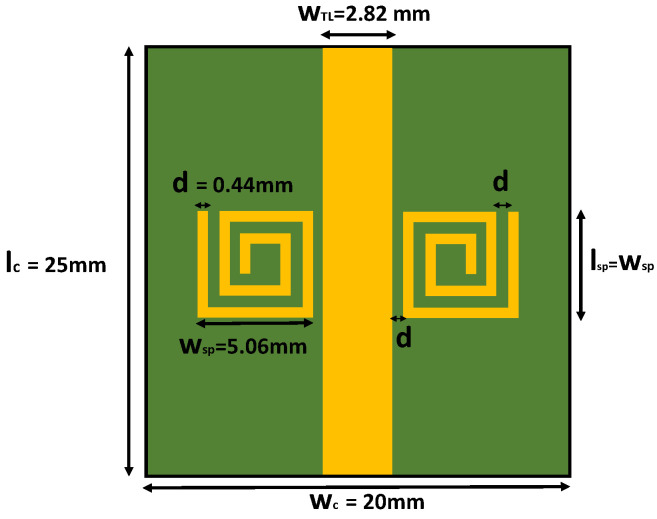
PCB layout of the sensor.

The SSR sensor designed for this paper (Figure 5) has two copper square spirals separated by a microstrip transmission line (TL), which excites both resonators through the incident microwave signal. The TL and the spiral resonators are located in the same plane (the top layer of the PCB). As the propagating mode of the microstrip TL is a quasi-TEM mode, there is a magnetic field perpendicular to the spirals surface that excites both resonators. The spirals produce a notch in the reflection coefficient of the TL at their resonant frequency. Two spiral resonators instead of one are coupled to the TL because it produces a deeper notch in the reflection coefficient. The copper TL and the spirals are etched in a copper-grounded PCB ($l_{c}=25\;\mathrm{mm}$, $w_{c}=20\;\mathrm{mm}$) made of FR-4 substrate with a relative permittivity $\varepsilon_{\mathrm{sub}}=4.5$ and a thickness of $h_{\mathrm{sub}}=1.5\;\mathrm{mm}$. The TL crosses the whole PCB with a width of $w_{TL}=2.82\;\mathrm{mm}$ and a thickness of $h_{TL}=0.35\;\mathrm{mm}$. The width was selected to adjust the characteristic impedance of the TL to 50 Ω, while the length of the TL was set to achieve good impedance matching at the resonant frequency of the spiral resonators. The center of both spirals is at the middle of the TL, and each spiral is separated from the TL with a distance of d=0.44mm. The same gap distance (*d*) is the width of each spiral arc as well as the gap between them. The spiral dimensions ($l_{sp}=w_{sp}=5.06\;\mathrm{mm}$) set the sensor resonance frequency at the vacuum reference.

**Figure 6:**
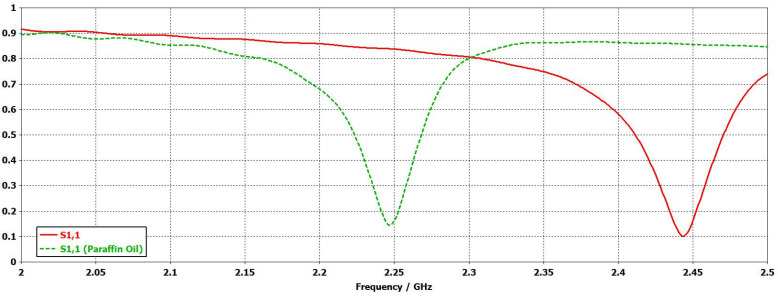
Simulated reflection coefficient $\left(|s_{11}|\right)$ of the sensor in vacuum (solid red) and covered with paraffin oil (dashed green).

The sensor was simulated by using the EM transient solver of CST Studio Suite (Dassault Systemes, Paris, France). Figure 6 shows the result of the EM simulations. The resonance frequency of the SSR-based sensor in vacuum is 2.444  GHz. The simulations also show the operation principle of the sensor. When the SSRs are covered with a material, there is a shift in the notch frequency related to the relative permittivity of the covering material. In the simulations, the SSRs were covered with paraffin oil (relative permittivity $\varepsilon_{\mathrm{par}}=2.13$ [28]) to show how the resonance frequency is shifted towards lower frequencies. In particular, the notch is shifted from 2.444 to 2.247  GHz when covering the sensor with paraffin oil.

**Figure 7:**
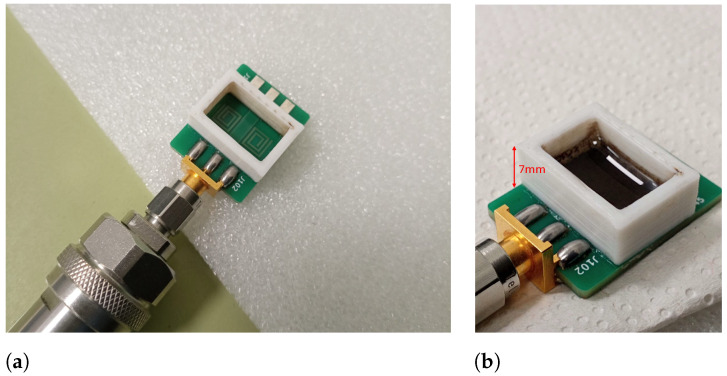
SRR sensor with the glued PLA pool: unloaded (**a**) loaded with a paraffin dispersion (**b**).

The sensor TL is welded to an SMA (SubMiniature A, RS Pro) connector, which is the interface for a vector network analyzer (VNA). The VNA (MS46122B, Anritsu, Atsugi, Japan) operates in the 1-port mode configuration to collect the reflection coefficient of the SSR, $\left|s_{11}\right|$, in the frequency domain. The VNA was calibrated in the 2.23 GHz to 2.46 GHz range to acquire the measurements, with a sampling buffer of 7000 points every 15 s. The SRR sensor presents a drift of 3 MHz around the resonance frequency due to the connection with the VNA port and experimental variability. In order to perform the measurements, a 3D-printed polylactic acid (PLA) container was glued with cyanoacrylate to the substrate to keep the dispersion of NPs in place (see Figure 7). The container was fabricated with a fused deposition modeling (FMD) 3-D printer (i3 Mega, Anycubic, Hong Kong, P. R. China). This container was not considered in EM simulations for simplicity. Thus, a small frequency shift is expected in the experimental results due to this fact and tolerances in the manufacturing process.

### 2.4. Measurement Protocol

We prepared nanoparticle dispersions by mixing Cu-NPs and Fe-NPs with liquid paraffin, a mineral oil mainly used for medical and cosmetical applications, with a low relative permittivity ($\varepsilon_{\mathrm{par}}=2.13$) [28]. Paraffin is a non-polar liquid that does not interact electrically with the nanoparticles, leaving gravity as the only acting force during sedimentation. In particular, the samples were prepared using a sonicator (Branston Digital Sonifier Model 450), which applied sound energy to agitate particles, resulting in homogeneous dispersions. The sonication was performed using a 50% tip amplitude for 15 min in pulsed mode, with a cycle of 15 s of activation and 5 s of rest. During this process, the tube containing the dispersion was submerged in water with ice to keep the nanoparticles’ temperature under control. Thus, each measurement is performed at a similar temperature in order to evaluate only how the nanoparticle type or concentration affects the sedimentation. After the sonication, a volume of 500 μL was placed inside the sensor PLA container for characterization. For comparison purposes, the measurements were performed for iron and copper nanoparticle dispersion at different concentrations. We expect that the sedimentation will assure a reproducible layer of nanoparticles directly proportional to the dispersion concentration.

The samples used for measurements started from a very saturated dispersion, around 20 mg/mL for Cu-NP and 15 mg/mL for Fe-NP. Afterward, the concentrations tested were reduced in steps of 5 mg/mL until a concentration near the paraffin reference signal was reached. On the other hand, all the fabricated Cu-NP mass (440 mg) was loaded in the sensor to characterize the dry nanopowder and compare its dielectric properties with the paraffin dispersion. In contrast, just half of the fabricated Fe-NP mass (170 mg) was analyzed due to its strong loss tangent. Nevertheless, a quantitative comparison of powder masses could not be achieved without a method to standardize compaction. Although the powder mass is a parameter directly proportional to the frequency shift, the critical factor is the compaction, which defines the internal volume of air and, consequently, the relative permittivity. For this reason, a direct permittivity measure of powder substances is hard to obtain, especially for resonant methods. However, additional solvents (500 μL) with known relative permittivity and loss tangent were measured to establish bounds for the NP dispersions dielectric properties (Table 2).

## 3. Results and Discussion

### 3.1. Dielectric Characterization of Nanopowders

**Figure 8:**
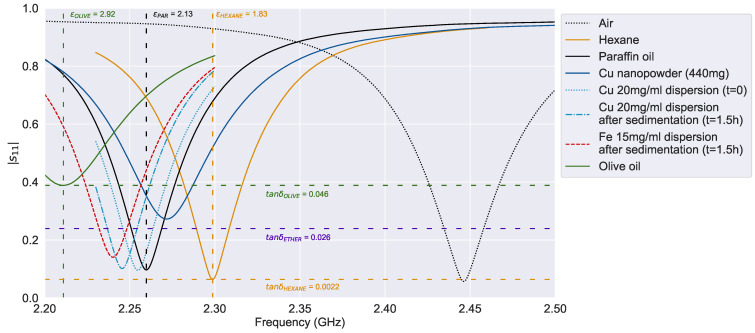
Sensor resonance curves for Cu nanopowders.

Figure 8 shows the resonance frequency shift and the amplitude reduction for several solvents, Cu-NPs paraffin dispersions, and air as the unloaded sensor reference. Moreover, the dry Cu nanopowder was also characterized before being dispersed in paraffin oil. This manually compacted sample shows a significant reduction in the resonance amplitude, which is an indicator of electromagnetic loss (tanδ), close to the amplitude measured for ethyl ether ($\tan\delta_{ETHER}$). In addition, its relative permittivity is bounded in the range between the values of paraffin oil [28] and hexane [29] (1.83 to 2.13 GHz). Indeed, paraffin oil has a larger relative permittivity but lower losses since the amplitude barely changes from the air reference or the hexane signal.

These insights show that Cu nanoparticles’ permittivity ($\varepsilon_{\mathrm{nanoCu}}$) is lower than paraffin permittivity ($\varepsilon_{\mathrm{paraffin}}$); therefore, the expected resonance frequency of the Cu dispersion before sedimentation (t = 0) should be slightly higher than liquid paraffin. Through sedimentation, the resonance frequency should increase, approaching the peak value of the dry Cu nanopowder sample. However, Figure 8 shows the opposite behavior: first, the Cu dispersion before sedimentation (t = 0) presents a resonance frequency lower than pure paraffin, and afterward, the sedimentation (t = 1.5 h) reduces the resonance frequency even more. Contrary to what was expected, these frequency shifts indicate that $\varepsilon_{\mathrm{nanoCu}}$ should be larger than $\varepsilon_{\mathrm{paraffin}}$.

The explanation for this behavior is that the Cu nanopowder is indeed a mixture of nanoparticles and air so that its effective permittivity is a weighted average between NPs and air permittivity. Since air permittivity is approximately equal to vacuum permittivity, the powder’s effective permittivity would be lower bound for the Cu-NP actual permittivity. Thus, depending on the apparent density of the powder, the effective permittivity would be a better approximation of the actual nanoparticle permittivity ($\varepsilon_{\mathrm{nanoCu}}$). The same rule is applied for the paraffin dispersion, but the medium relative permittivity is now 2.13 instead of 1. In this case, since $\varepsilon_{\mathrm{paraffin}}$ is closer to $\varepsilon_{\mathrm{nanoCu}}$ than $\varepsilon_{\mathrm{air}}$, the dispersion effective permittivity is closer from the actual $\varepsilon_{\mathrm{nanoCu}}$. Therefore, through sedimentation, the dispersion effective permittivity approaches this value when the layer of nanoparticles is formed on the sensor surface, and the volume of paraffin is reduced in the near-field sensed volume.

**Figure 9:**
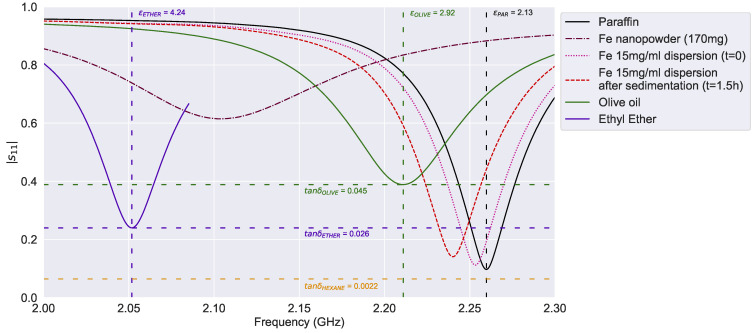
Sensor resonance curves for Fe nanopowder.

The effect of compaction can explain the permittivity of Cu-NP powder, and this explanation can also be extended to Fe-NP. Nevertheless, as is depicted in Figure 9, both the relative permittivity and the loss tangent are significantly greater than dry Cu nanopowder (Figure 8). In addition, this acute amplitude loss could be related to the ferromagnetic properties of Fe-NP. Thus, the dry Fe nanopowder response is also a lower-bound estimation, but due to its dielectric properties, it is measured beyond the paraffin response and does not seem contradictory, as happened with the Cu nanopowder lower-bound. On the other hand, as expected, the frequency response of Fe-NP dispersion is relatively close to Cu-NP dispersion since the main component of both is the liquid paraffin (Figure 9). However, the slight differences between both dispersions are due to the dielectric properties of each NP. Indeed, even if the concentration of Fe-NP (15 mg/mL) is lower than the Cu-NP concentration, the frequency shift is more pronounced for NP-Fe. It must be noted that the Fe-NP 20 mg/mL concentration was over-saturated, and it was diluted to 15 mg/mL for this comparison analysis.

Despite these differences between paraffin dispersions, their dielectric response is within the boundaries of the reference solvents. While the relative permittivity varies between the values of paraffin oil [28] and olive oil [30] ($\varepsilon_{r}=$ 2.13 to 2.92), the loss tangent is limited by the hexane [29] and ethyl ether [31] electromagnetic losses (tanδ= 0.0022 to 0.026). Likewise, the dielectric studies performed by Mergos et al. show similar slight permittivity differences between paraffin dispersions [28].

### 3.2. The Effect of Concentration on the Nanoparticle Sedimentation

**Figure 10:**
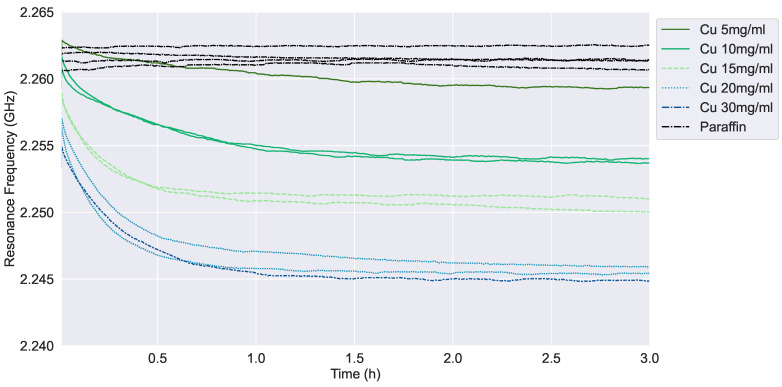
Resonance frequency versus time in the case of different concentrations of Cu nanopowder.

Figure 10 shows that the resonance frequency for paraffin remains constant over time; consequently, its value can be taken as a baseline for comparison purposes, which reveals a negligible sensor drift around 3 MHz. All Cu-NPs dispersions show a steep decrease in the resonance frequency during the first hour; afterward, the effect of sedimentation on the sensor is asymptotically reduced up to a steady state value. When the concentration of the Cu dispersion is lower, e.g., see the cases considering 5 and 10 mg/mL concentrations, the initial resonance frequency is within the range of liquid paraffin signals, proving that the dielectric effect of nanoparticles in the bulk liquid is minimal. Indeed, after 3 h the Cu 5 mg/mL layer value is almost below the detection limit of the sensor. Therefore, for lower concentrations, we expect that the formed NPs layer would not have enough impact on the permittivity measured by the sensor. However, the variation of resonance frequency due to the sedimentation is still noticeable even though the resonance frequencies for both dispersions overlap with the paraffin one.

The sedimentation curve is steeper for higher concentrations, such as Cu 15 or 20 mg/mL, in comparison to Cu 5 or 10 mg/mL. Moreover, the agglomeration between the particles, which is not studied within this paper, could play a relevant role in determining a quicker sedimentation at higher NPs concentrations. It should be noted that Cu 30 mg/mL was over-saturated after sonication, and sedimented particles were observed at the bottom of the tube. For this reason, the Cu 30 mg/mL signal is quite similar to Cu 20 mg/mL, which is very close to saturation.

**Figure 11:**
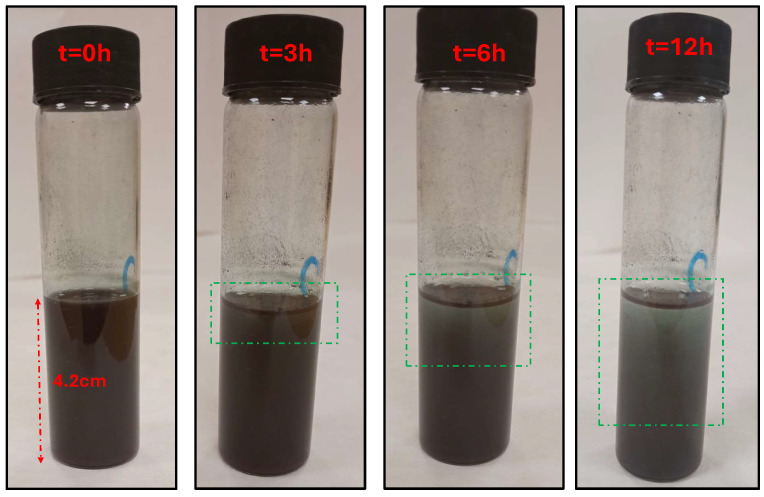
Cu-NP dispersion (15 mg/mL) at different times after sonication; the green boxes show the liquid volume that is starting to become transparent due to sedimentation.

In contrast, the Cu-NP sedimentation within the sonication tubes shows less noticeable changes in the 3-h interval (Figure 11). Indeed, the complete sedimentation time is considerably longer; even after 6 h, most of the NP are still dispersed in the paraffin. This long operation time has been the main drawback of sedimentation techniques for NP characterization, such as measuring the bulk fluid with absorption spectroscopy [32]. However, the measured sedimentation profiles indicate that the SRR sensor can achieve a faster detection time since it aims to measure only the bottom thin layer made by the first NP sedimented. In combination with the high sensitivity in the near-field from the SRR surface, the sensor does not require waiting until the sedimentation is finally complete.

**Figure 12:**
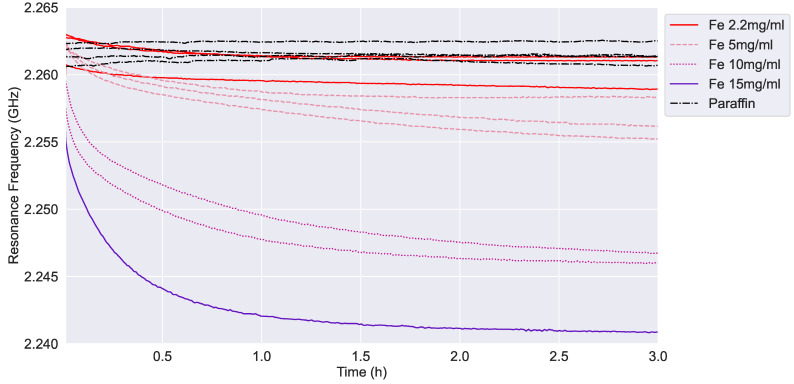
Resonance frequency versus time in the case of different concentrations of Fe nanopowder.

On the other hand, Figure 12 shows a similar sedimentation dynamic in the case of Fe dispersion samples. The main difference is that Fe dispersion requires a lower concentration to achieve a resonance frequency similar to Cu sedimentation curves. These results match with the dielectric characterization of nanopowders. For example, Fe 10 mg/mL dispersion has a similar resonance frequency to Cu 15 mg/mL, and the previous detection limit is reduced from Cu 5 mg/mL to Fe 2.2 mg/mL. In addition, Fe 15 mg/mL achieves a higher frequency than Cu 20 mg/mL, with both concentrations being the saturation limit of each nanopowder. This difference in solubility is directly related to the properties and can be determined with the SSR sensor.

**Figure 13:**
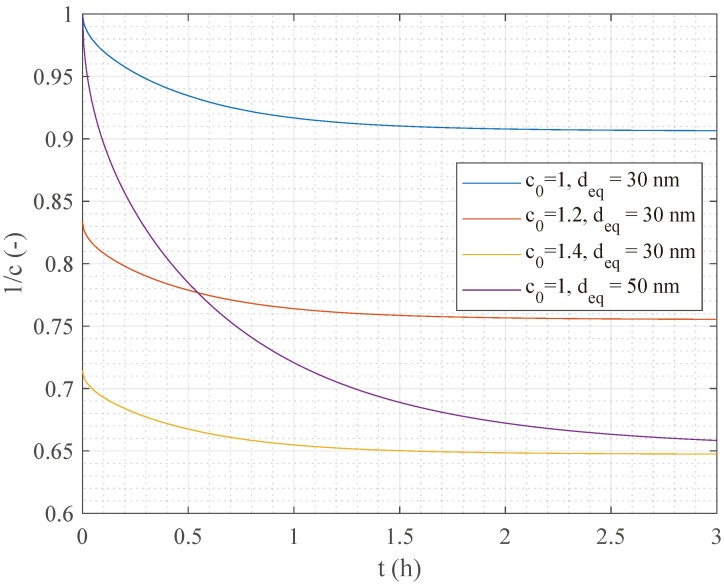
Example of 1/c calculated at x=0.1mm. c(t) is obtained by solving the Mason–Weaver equation with T=300 K, and various initial concentration and particle diameters.

Both Figure 10 and Figure 12 show a decay of the resonance frequency measured by the SSR sensor as a function of the time, which is consistent with the conceptional principle described in Section 2.1. We recall that, according to Equation (1), *c* changes vs. *t*, finally reaching an equilibrium distribution for t→∞. We interpret this occurrence as the deposition of NPs to form a growing layer on the sensor surface. As the layer grows, the average relative permittivity in the sensor sensitive region increases too, thus decreasing the value of the measured resonance frequency. Looking at Figure 10 and Figure 12, we can observe a qualitative agreement vs. *t* between *f* and 1/c, presented in Figure 13.

In particular, the higher is the initial concentration, the lower the equilibrium resonance frequency is obtained. However, experimental results indicate a faster frequency decay right from the onset of the process as a function of the initial NPs concentration. We also notice that a faster decay appears by simulating larger particles utilizing the Mason–Weaver model (see Figure 13). Hence, we infer that such a decay could arise due to particle clustering, which is likely influenced by the solid phase concentration [33]. Although Equation (1) model is not able to take into account such a phenomenon, it offers a simple yet reliable basis for discussion, substantially contributing to the validation of our results. In this context, the SSR sensor has demonstrated the capability of carrying out relevant information on the sedimentation dynamic, and hence of the NPs properties and their concentration, through the analysis of the medium resonance frequency, thus highlighting the potential of the proposed technique for characterizing metallic nanopowders.

## 4. Conclusions

We studied the effect of sedimentation of NPs in a viscous media through the measurement of the electrical characteristics of the paraffin-NPs dispersion over time through an MW sensor. The sensor is based on two SSRs coupled to a printed TL, and it operates in the MW regime. A resonant notch is observed in the reflection coefficient of the sensor. The resonant frequency depends on the materials deposited over the sensor surface. In particular, several dispersed solution samples were prepared by employing Cu- and Fe-based nanoparticles and paraffin oil. The NPs used in the experiments have been produced via a wire explosion process. During the experiments, we observed a reduction in resonance frequency and signal reflection over time, both attributed to the impact of sedimentation on the dispersed solution properties. Moreover, all the sedimentation profiles converge after 3 h, suggesting that the nanoparticle layer measurable by the SRR is completed at that time. Therefore, the methodology proposed in this research is a relative fast technique to characterize nanoparticles. Indeed, our investigation enabled the differentiation of distinct patterns arising from varied types and concentrations of nanopowders within the solution. Therefore, we used the Mason–Weaver model as a basis for the discussion of the results. These insights point to the possibility of using MW sensors to characterize the outcome of nanopowders production. Although the results are indeed quite tied to the structure of the specific sensor and require a uniform preparation of the sample, the repeatability is high, and the differences between different types or concentrations of NPs are clearly detectable with few parameters like time constant and final steady-state value of the resonance frequency evolution.

The results of this research also suggest that a concentration around 10 mg/mL is the most optimal for characterizing Fe-NP and Cu-NP due to a compromise between dielectric response and product waste. Moreover, measuring the sedimentation with the SRR sensor could reveal other NP characteristics beyond dispersion concentration or chemical composition. For example, the particle size could be estimated since the sedimentation time depends on the gravitational pull, which will be stronger for larger particles. In addition, the microwave sensor technology could be improved by implementing low-cost electronics and machine learning techniques [34], thus reinforcing its suitability as a characterization method. In conclusion, the methodology proposed in this research is a solid and faster alternative for characterizing nanoparticles and avoiding complex instrumentation.

## Figures and Tables

**Table 1:** Main parameters of the dimensional distribution of the analyzed samples.

	Minimum $d_{\mathrm{eq}}$	Median $d_{\mathrm{eq}}$	Average $d_{\mathrm{eq}}$	Maximum $d_{\mathrm{eq}}$	
Cu-NPs	29	35	52	204	(nm)
Fe-NPs	6	28	36	228	(nm)

**Table 2:** Dielectric properties of several solvents at microwave frequencies.

	$\varepsilon_{r}$	tanδ	Reference
Paraffin oil	2.13	0.001	[28]
Hexane	1.83	0.0022	[29]
Olive oil	2.92	0.046	[30]
Ethyl Ether	4.24	0.026	[31]

## Data Availability

The raw data supporting the conclusions of this article will be made available by the authors on request.
